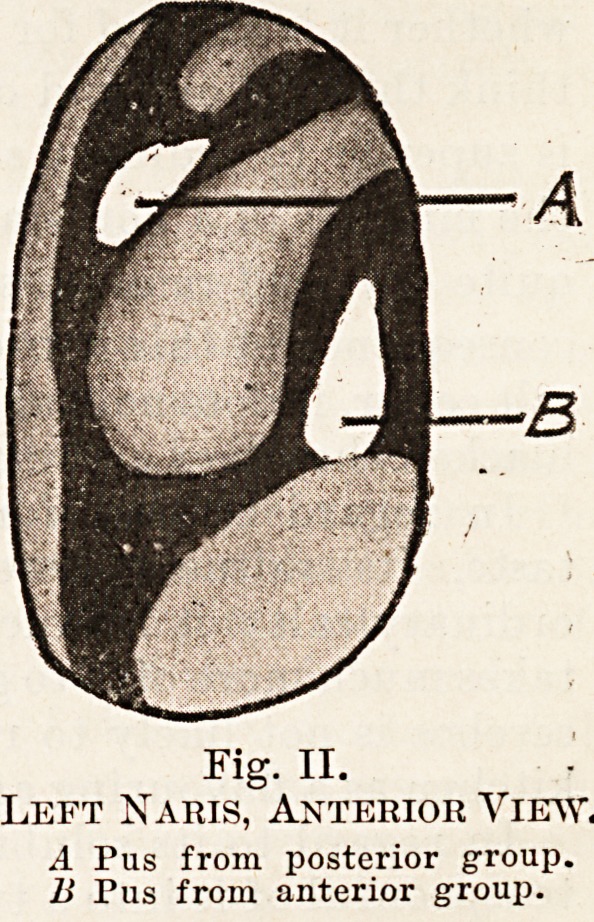# Nasal Sinus Suppuration

**Published:** 1907-06-15

**Authors:** 


### LARYNGOLOGY AND RHINOLOGY.

### NASAL SINUS SUPPURATION.

SUPPURATION in the accessory cavities of the nose is very common and may produce far-reaching effects; many of these cases are quite obvious, others are very obscure and may easily escape detection. The trouble usually results by infection from the nose, from an ordinary coryza or from the rhinitis of one of the specific fevers, especially influenza; even the antrum is more often infected in this way than from carious teeth. The micro-organisms found are the ordinary pyogenic cocci, the pneumococcus, the bacillus influenzæ, etc.; bacilli characteristic of dental caries are only occasionally present, and the tubercle bacillus is quite rare.

There are three principal symptoms, namely, pain, discharge, and a variety of distant effects; and the cases may roughly be divided into three classes—(1) closed, (2) open, and (3) latent, according as one or other symptom predominates. The latent cases are not uncommon; there is no localising pain, though there may be indefinite headache, and the patient does not complain of discharge, and may even deny it when questioned; but a considerable quantity of pus passes nevertheless down the throat, and is swallowed, although several examinations may be necessary before any is found in the nose. The results include pharyngitis, laryngitis, bronchitis, dyspepsia, anæmia, and general ill-health; while the combination of loss of health, cough and purulent expectoration may arouse a suspicion of phthisis. Serious complications, such as meningitis and cerebral abscess, may result from extension of inflammation in the latent, as well as in other forms of sinus disease.

In the "open" form of suppuration the principal symptom is purulent discharge, the position of which in the nose gives most valuable aid in determining the affected sinus; a unilateral discharge in an adult patient is nearly always due to sinus disease. The nasal sinuses are divided into anterior and posterior groups. The former are the maxillary antrum, frontal sinus and anterior ethmoidal cells, opening into the middle meatus beneath the middle turbinal by a groove or gutter called the *hiatus semilunaris*; the latter are the sphenoidal sinus and posterior ethmoidal cells, which open far back above the middle turbinal. Pus from these sinuses runs chiefly into the throat, while the discharge from the anterior group comes mostly to the nostrils; this rule is by no means absolute, for a sharply curved middle turbinal will direct matter backwards into the throat, and the antrum often has an accessory ostium situated above the middle turbinal. On examining the nose the pus may be seen in the neighbourhood of one of the orifices; if the nose be full of pus this should be wiped, not syringed away, and its reappearance watched for. To see well into the hiatus semilunaris it may be necessary to gently press the middle turbinal away with a probe; if pus be seen here it must come from a sinus of the anterior group, whereas pus from the sphenoidal or posterior ethmoidal cells shows itself between the middle turbinal and the septum. Now examine the posterior nares with the rhinoscope mirror; if discharge be seen beneath the middle turbinal it comes from the anterior group, but pus in the choana above the middle turbinal is one of the surest signs of sphenoidal or posterior ethmoidal disease. We have now to differentiate empyæma of the antrum from that of the frontal sinus; the antral ostium is situated at the lower and posterior end of the hiatus, while the frontal opening is at its upper anterior extremity; therefore, pus far forward and high up in the hiatus is strongly suggestive of frontal sinusitis. A curved probe can often be passed into the frontal sinus, and usually if the anterior end of the middle turbinal be removed, and if pus be seen to flow along it the diagnosis is practically concluded. The antral opening is near the roof of the cavity, consequently pus will often flow from an antral empyæma with a gush on bending the head forwards and to the opposite side; a frontal sinus discharges best in the upright posture. In difficult cases the antrum may be tapped with a fine trocar and cannula from the inferior meatus of the nose under cocaine, and its contents washed out; of course the nose must first be carefully cleaned of all discharge. Before doing this, however, transillumination by means of a small lamp in the mouth should always be tried, any artificial denture being first removed. The *tâche* of light which corresponds to the antrum is a crescentic area just below the infra-orbital margin; the pupil should also appear red and the patient should be conscious of a sensation of light. If the antrum light up clearly it is certain that it does not contain pus, but if it be dark it by no means follows that it is diseased, though if it be dark upon the affected side and quite clear upon the other the presumptive evidence is strong. The frontal sinus can also be transilluminated by applying the light to its floor through a funnel, but the variability in size of these cavities makes a negative result of little value. When the posterior group is affected the distinction of ethmoidal and sphenoidal disease is often difficult and, indeed, they are fre-

Fig. I.
LEFT NARIS, ANTERIOR VIEW.
*A* Pus from posterior group.
*B* Pus from frontal sinus.
*C* Pus from antrum.

Fig. II.
LEFT NARIS, ANTERIOR VIEW.
*A* Pus from posterior group.
*B* Pus from anterior group.

quently diseased together; it is usually necessary to remove the middle turbinal, after which the affected cells can be probed and the pus followed to its origin.

If the orifice of a sinus be blocked by inflammatory swelling pain becomes a prominent symptom, for the contents of the cavity collect under pressure. This, the "closed" variety, includes the acute cases, for in them there is always some tension; acute exacerbations are very common in the course of an ordinary chronic or "open" sinusitis. The opening is seldom completely obstructed, and there is generally some discharge into the nose. The resulting pain is often characteristic, gradually increasing in severity until the discharge begins to flow, when it is at once relieved. Its localisation varies with the sinus affected and, though by no means constant, has some diagnostic value. Thus, in antral disease it is usually felt in the infra-orbital and malar region and radiates to the temple; in frontal sinusitis it is often felt high up on the forehead near the coronal suture; in ethmoiditis it is deep in the head and behind the eyes; and in empyæma of the sphenoidal sinus it is usually situated at the back of the head and neck and also behind the eyes. In all cases pain along the path of the supra-orbital nerve often occurs; it appears to be due to pressure on the middle turbinal, which is inflamed and swollen by irritation of the pus passing over it. It is important to remember that supra-orbital pain is very common in antral disease, and is in no way diagnostic of frontal sinusitis. The localisation of tenderness on pressure is of more value; in antral cases it is often about the base of the nasal process of the superior maxilla, while in frontal sinusitis it is best elicited by pressure on the thin inferior wall at the upper and inner angle of the orbit.

## Figures and Tables

**Fig. I. f1:**
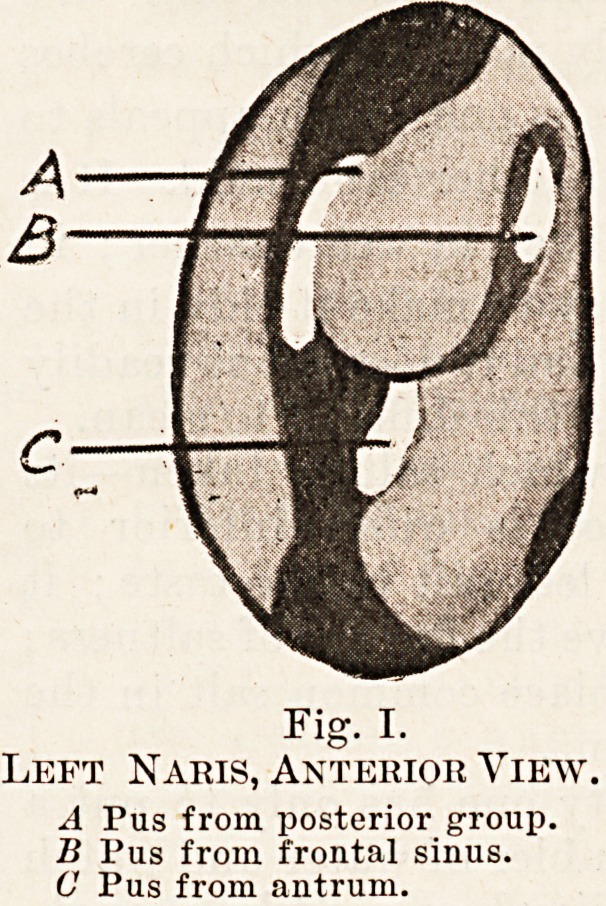


**Fig. II. f2:**